# A CD40-targeting peptide, OPT501, modulates inflammation in canine diabetes mellitus improving clinical outcomes

**DOI:** 10.3389/fimmu.2026.1759373

**Published:** 2026-02-24

**Authors:** Gisela M. Vaitaitis, Dan M. Waid, Christina Sharkey, Steve Sharkey, Tracy L. Webb, Craig Webb, David H. Wagner

**Affiliations:** 1 Department of Medicine, The University of Colorado Anschutz Medical Campus, Aurora, CO, United States; 2 Op-T LLC., Aurora, CO, United States; 3 Montclair Animal Clinic, Denver, CO, United States; 4 Department of Clinical Sciences, Colorado State University, Fort Collins, CO, United States

**Keywords:** canine diabetes mellitus, CD40-targeting peptide, inflammation, OPT501, pilot study, treatment

## Abstract

**Introduction:**

The etiology of Canine Diabetes Mellitus (CDM) is poorly understood but findings like increased CD3^+^CD4^+^CD40^+^ pathogenic effector T cells (Th40 cells), support an autoimmune contribution. Despite insulin supplementation and possible residual C-peptide in CDM, many dogs remain severely dysglycemic, with weight loss, cataracts, and chronic and recurrent infections. In human and murine autoimmune disease, CD40-CD154 acts as a prominent inflammation driver but targeting that interaction, and others, with antibodies has been plagued by complications such as thrombotic emboli or immunosuppression. We developed small peptides that target CD40 and that are not accompanied by the side effects attributed to antibodies. In mice, such a peptide prevented and reversed type 1 diabetes.

**Methods:**

We utilized a CD40-targeting peptide, OPT501, to treat CDM dogs via an intravenous or subcutaneous route and followed their disease status and clinical outcomes as well as their inflammatory status.

**Results:**

Treatment with OPT501 significantly decreased pathogenic Th40 cells, the systemic inflammatory index, and fructosamine (an analog to human HbA1c). This led to lowered insulin requirements while improving blood glucose regulation. OPT501 also significantly reduced cholesterol and alkaline phosphatase, and significantly increased plasma C-peptide, a measure of endogenous insulin production.

**Conclusions:**

This pilot and proof-of-concept study demonstrates that targeting CD40 with a peptide is feasible and impacts the inflammatory status of the recipient CDM dogs, with improved disease management as a result. The C-peptide result is consistent with preservation of islet beta cell health and function. These data support translation of a CD40 targeting peptide approach to human type 1 diabetes.

## Introduction

Canine diabetes mellitus (CDM) afflicts up to 1.2% of companion dogs in the U.S. with some breeds showing a higher predisposition (1). The incidence of CDM has been reported to have risen 79.8% since 2006, which mirrors the sharp increase observed in human T1D. The impact of CDM on dogs and owners is profound with a reported euthanasia rate of 10% at diagnosis and another 10% euthanized within the following year (1). Although the etiology remains poorly understood, CDM results in hyperglycemia related to extensive beta cell loss (2). Persistent hyperglycemia and insulin dependency are common traits in CDM (1). While insulitis, i.e., infiltration of pancreatic islets by peripheral immune cells, has been well described in human type 1 diabetes and in research models, it has rarely been reported in CDM (1). One possible explanation is that insulitis in human type 1 diabetes predominantly occurs in younger subjects (<15 years old) with acute disease (< 1 year) (3). If a similar pattern exists in dogs, the opportunity to observe insulitis would be missed when studying pancreata from dogs that died well after disease onset.

One hallmark linked with human type 1 diabetes is the presence of autoantibodies (Aabs) (4), but the Aabs associated with human type 1 diabetes have been difficult to detect in CDM (1, 5). However, alternate autoantigens in CDM compared to human type 1 diabetes have been reported (6). Disease management in human type 1 diabetes is measured by the levels of glycated HbA1c. In CDM patients, fructosamine is an analogous assessment of diabetic control (7). Fructosamine occurs when amino acids are glycated by free glucose and thus is a reflective measure of glycemia. Fructosamine levels in all diabetic dogs in our recent study were significantly higher than the canine normal range, consistent with poor disease management (8). Other inflammatory indices, such as the systemic (SII) and chronic inflammation (CII) indices, were significantly elevated in CDM dogs compared to healthy controls (8). These findings suggest that inflammatory dysregulation is a factor in CDM.

We recently reported that CDM dogs have a significant (p< 0.0001) expansion of pathogenic effector CD4 T cells that express CD40, termed Th40 cells, compared to healthy controls (8). Th40 cells were originally described in the non-obese diabetic (NOD) mouse model of autoimmune diabetes (9), and translational studies showed Th40 cell expansion within peripheral blood mononuclear cells of human type 1 diabetes, including Stage 1, Stage 2, and Stage 3 human subjects (10, 11). Th40 cells proved necessary and sufficient to transfer type 1 diabetes in the NOD mouse model (9). Th40 cells from CDM dogs produced the proinflammatory cytokines IL-6, TNFα, and IFNγ (8), similar to Th40 cells in the NOD mouse and human type 1 diabetes (11, 12), suggesting an autoimmune link.

In the NOD mouse model of type 1 diabetes, blocking CD40-CD154 signaling with anti-CD154 antibody prevents disease onset, but only when administered at less than nine weeks of age (13). When the monoclonal antibody strategy was used in human disease, life-threatening adverse events (i.e., thrombotic emboli) occurred (14, 15). We developed a different strategy to target the CD40-CD154 dyad utilizing small peptides (12, 16) that target CD40 to modulate rather than block signaling (17). The peptides include a region of CD154 that was shown by crystallography and mutational analysis to interact with CD40 and have a core sequence **K**G**YY** (lysine, glycine, tyrosine, tyrosine; amino acids in bold were required for CD40 interaction) (18). One version of these peptides, OPT101, was able to prevent disease onset in the NOD mouse model of type 1 diabetes (12). Unlike the monoclonal antibody approach, that peptide was able to reverse overt diabetes in new onset disease in 56% of treated mice (12). We performed structure-activity relationship (SAR) studies by creating a series of peptides where single amino acids were sequentially replaced by glycine. In contrast to the crystallography studies, the SAR studies revealed that five amino acids in the OPT101 sequence are involved in disease prevention (12). OPT101 has recently undergone safety/tolerability testing, completing Phase 1a and Phase 1b clinical trials (ClinicalTrials.gov study # NCT05428943) in healthy human control subjects (Phase 1a) as well as in human type 1 diabetes (Phase 1b) subjects, with excellent tolerability and safety, and only mild to moderate adverse events.

Working from the understanding that CDM has an autoimmune etiology, we developed a canine version of the peptide, OPT501. Here we show the results of a pilot study treating CDM dogs with the OPT501 peptide.

## Materials and methods

### Study design

This was an open label, non-blinded pilot study of 10 CDM dogs receiving the study peptide, OPT501, either intravenously or subcutaneously for up to 24 weeks. As a pilot study, there were no placebo or control groups. The study (I-013432) was approved by the Center for Veterinary Medicine, a branch within the US Food and Drug Administration. Clinical and disease management changes were recorded, and Th40 cell levels determined before, during, and after treatment. The dogs were housed at home with their owners and no dietary restrictions, instructions regarding physical activity, or other restrictions were in place. That is, the owners treated and fed their dogs per their normal schedules.

### Subject recruitment

The study was reviewed and approved by the Colorado State University Clinical Review Board (protocol #640). Ten CDM subjects were recruited through the Colorado State University Veterinary Teaching Hospital in Fort Collins, CO, or at the Montclair Animal Clinic in Denver, CO. All subject owners were consented prior to enrollment of their dog. All experiments were carried out in accordance with ARRIVE guidelines and relevant regulations.

### Treatment peptide OPT501

OPT501, a 15-amino acid peptide was synthesized as an acetate salt at AmbioPharm, Inc., North Augusta, SC, in a cGMP facility. The certificate of analysis confirmed that the peptide was 97% pure. It was received as a lyophilized powder and was stored at -80°C until use. For short term storage, up to 3 months, the powder was stored at -20°C. For intravenous (i.v.) and subcutaneous (s.c.) administration, OPT501 was dissolved in sterile Phosphate Buffered Saline (PBS), pH 7.2.

### Treatment

Treatment of CDM dogs was preceded by a safety and tolerability study done through a CRO, WuXi Biologics, where OPT101 (human version of the drug) was administered via i.v. infusion over 30 minutes to both rats and healthy dogs. OPT101 was well tolerated, and only moderate adverse events (injection site reactions related to histamine release; the higher doses were associated with decreases in blood pressure and heart rate, which returned to normal 1 hour after the end of infusion) were observed at doses 25–50 times higher than the doses used in this study.

Subjects treated with OPT501 via the i.v. route received 30-minute infusions of 1–2 mg/kg on Monday, Wednesday, and Friday the first week then once weekly for 8 weeks. Subjects treated with OPT501 via the s.c. route received a dose of 2.0 mg/kg on Monday, Wednesday, and Friday the first week followed by 2.0 mg/kg twice weekly, Monday/Friday, or 4 mg/kg once per week for up to 24 weeks. Some subjects received OPT501 s.c. at a dose of 4 mg/kg twice weekly for up to 24 weeks. Some dogs continued to receive weekly s.c. injections for up to 18 months. Adverse events: Other than injection site irritation with s.c. dosing, no OPT501 related adverse events were observed in the CDM dogs. Changes in insulin dosage were done under advisement of the attending veterinarian.

### Clinical analysis including complete blood count, and blood chemistry panels

Venous blood was drawn into 2 ml heparinized blood collection tubes for preparation of peripheral blood mononuclear cells (PBMC). Blood was drawn into EDTA tubes for complete blood count (CBC) and red top tubes for chemistry panels. CBC and chemistry panels were processed at each veterinary clinic’s associated laboratory service (CSU: Veterinary Diagnostic Laboratory; Montclair: Antech, Inc.).

Normal ranges for the CSU Veterinary Diagnostic Laboratory are: Lymphocytes – 1000-4,800/µl; Neutrophils – 2.6–11 x 10^3^/µl; Platelets – 200–500 x 10^3^/µl; Alkaline Phosphatase – 15–140 IU/L; Alanine Aminotranferase – 10–90 IU/L; Cholesterol – 130–300 mg/dl; Fructosamine – 210-350 µmol/L; Glucose – 70–115 mg/dl.

Normal ranges for the Antech laboratory are: Lymphocytes – 690-4,500/µl; Neutrophils – 2.06-10.6 x 10^3^/µl; Platelets – 170–400 x 10^3^/µl; Alkaline Phosphatase – 5–131 IU/L; Alanine Aminotranferase – 12–118 IU/L; Cholesterol – 92–324 mg/dl; Fructosamine – 136-350 µmol/L; Glucose – 70–138 mg/dl.

Systemic Inflammatory Index calculation: The Systemic Inflammatory Index (SII (19, 20);) is derived by multiplying the total number of neutrophils and platelets then dividing by the total number of lymphocytes ((neutrophils x platelets)/lymphocytes).

### PBMC preparation, cell staining, and analysis

PBMCs were prepared using Ficoll Paque Plus (Cytiva, Marlborough, MA) according to the manufacturer protocol. Resulting PBMC were stained in PBS (Cytiva, Marlborough, MA) containing 0.5% bovine serum albumin and 2 mM EDTA (both from Sigma-Aldrich, St. Louis, MO). Cells were stained with anti-dog CD4, anti-dog CD3 (Bio-Rad, Hercules, CA; cat# MCA1038APC and MCA1774F, respectively), and anti-human CD40 (produced in-house; clone G28-5). Analysis was done using FlowJo software (Becton Dickinson, Franklin Lakes, NJ). Cells were first gated on live cells then on CD3. CD4^+^CD40^+^ percentages of total CD4^+^ in the CD3 gate are reported for dogs as previously described [8].

### Mouse and human Th40 level determination

All human subjects, female and male, >18 years old, with or without stage 3 type 1 diabetes, were recruited at the Barbara Davis Center for Diabetes and gave consent under Colorado Multiple Institutional Review Board protocol no. 01-384. The duration of disease in the T1D subjects was 6 weeks to 40 years. Pre-T1D subjects were part of a TrialNet study (10) and met the criteria (high-risk HLA and/or being a first degree relative of a subject with T1D) for inclusion.

Non-obese diabetic (NOD) and Balb/c mice (Taconic Biosciences, Germantown, NY) were housed at an AAALAC-approved facility. All animal experiments were performed under an IACUC-approved protocol (#00529) and adhered to the NIH Public Health Service Policy on Humane Care and Use of Laboratory Animals.

Blood was drawn from mice into heparinized syringes and from humans into heparinized blood collection tubes. PBMC preparation, cell staining, gating and analysis was done exactly as described above except that the following antibodies were used: Anti-human CD3, cat# 130-113-134 (Miltenyi Biotec, Waltham, MA), anti-human CD4, cat# 25-0049-T100 (Cytek Biosciences, Fremont, CA), anti-human CD40, clone G28-5 (produced in-house), anti-mouse CD3, cat# 12-0031-82 (Thermo Fisher Scientific, Waltham, MA), anti-mouse CD4, cat# 130312 (BioLegend, San Diego, CA), and anti-mouse CD40, clone 1C10 (produced in-house).

### C-peptide ELISA

C-peptide was measured in plasma retrieved from PBMC separation utilizing an ELISA kit from MilliporeSigma (St. Louis, MO; cat# EZCCP-47K). This kit did not state a normal range for dogs. The samples were collected without instructions for fasting or postprandially and the data is therefore exploratory as the values can vary with/without meals.

### Three-dimensional peptide folding

Three-dimensional peptide folding was done using the on-line peptide folding prediction program PEP-FOLD3, available at the RPBS web portal (https://mobyle.rpbs.univ-paris-diderot.fr) (21).

### Statistical analysis

Statistical analysis was performed using the Prism program from GraphPad (San Diego, CA) and the data is expressed as means (SEM). T-tests, unpaired or paired, and one-way ANOVAs were used as indicated in the figure legends and p-values of < 0.05 were considered significant.

## Results

### A canine version, OPT501, of drug product OPT101

**Figure 1:**
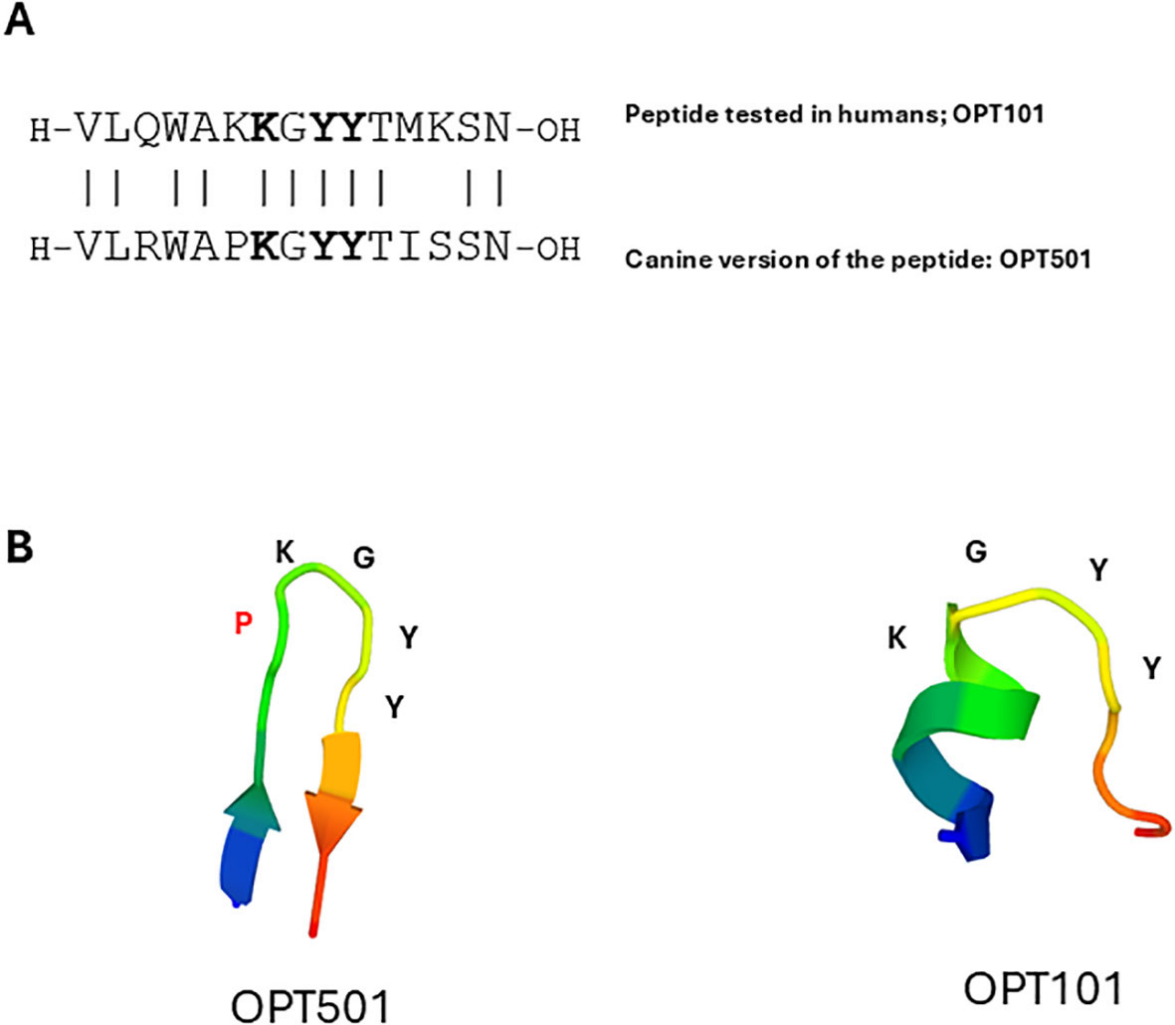
OPT501 is similar to OPT101. **(A)** A comparison of OPT101 and OPT501, showing the KGYY motif in bold. **(B)** Representations of the folded peptide structures of OPT101 and OPT501.

OPT101 for human use was derived from the known interaction site between CD154 and CD40 to include three of the five amino acids described by crystallography to be critical for CD40 interaction (12). Canine CD154 and murine CD154, the sequence from which OPT101 was derived (12), are both 260 amino acids in length. The peptide, OPT501, was designed as a 15-mer analogous to the drug product OPT101 (**Figure 1A**). Within the canine total protein sequence, the 15-mer was generated by beginning at Valine (V) 136 and continuing through Asparagine (N) 150 (**Figure 1A**). The **K**G**YY** shown by crystallography to be involved in the binding of the CD154 protein to CD40 was included (**Figure 1A**; bold amino acids). The canine sequence differs at four amino acids when directly compared to OPT101: a Glutamine (Q) to Arginine (R) substitution, a Lysine (K) to Proline (P) substitution, a Methionine (M) to Isoleucine (I) substitution, and a Lysine (K) to Serine (S) substitution (**Figure 1A**). In prior studies, specific residues within OPT101 were assessed for their contribution to peptide activity in disease-relevant models (12). Crystallography data reported by Bajorath et al. (18) indicated that Tyrosine in positions 9 and 10 (positions 144 and 145 in the whole protein) were crucial for CD154 and CD40 interaction. However, our functional peptide data showed that Tyrosine in position 9 is crucial for biological function in prevention of diabetes while Tyrosine in position 10 is dispensable (12) (**Figure 1A**). These observations identify **K**G**Y** as a conserved core motif within this region, which the canine CD154 sequence contains (**Figure 1A**). Given the amino acid differences, we compared the likely models of OPT101 and OPT501 using the PEP-FOLD 3 program for three-dimensional peptide modeling (21). The models predict that OPT101 adopts a helical segment that is not likely in OPT501, whereas the models predict that the Proline in OPT501 may facilitate a tighter fold of the peptide (**Figure 1B**; Proline in red). However, the models predict a **K**G**YY**-containing planar region in both peptides consistent with potential interaction with CD40 (**Figure 1B**).

### Subject demographics

CDM subjects were recruited, owners consented, and the demographic data is presented in **Table 1**. All participants could be classified as mid-life or older (median: 9.5 years, range: 6–13 years). Disease duration ranged from one month to 48 months (**Table 1**). The two patients at one month since diabetes onset were considered new onset. All subjects experienced unregulated blood glucose despite on-going insulin treatment with weekly averages ranging from 350 to 450 mg/dl glucose. Unlike human type 1 diabetes patients, the CDM patients, all of which were treated with Vetsulin™, had difficulty regulating glucose even on high dose insulin. At diagnosis blood glucose was high with glucosuria and three dogs had urine ketones. Urine ketosis follows ketonemia in poorly regulated diabetes and can lead to diabetic ketoacidosis, which requires close monitoring and intensive care. Five of the dogs (50%) had cataracts.

**Table 1:** Subject demographics and disease duration.

Breed	Sex	Age	Time Diabetic
Terrier mix	Male	9 years	6 mos.
Golden	Female	11.2 years	48 mos.
Border collie	Female	6 years	3 mos.
Yorkshire	Male	12.2 years	1 mo.
Giant Schnauzer	Female	10 years	1 mo.
Mix	Male	8.5 years	42 mo.
Collie	Female	13 years	6 mos.
Cocker spaniel	Female	8 years	9 mos.
Poodle mix	Male	11.6 years	12 mos.
Chihuahua	Female	8.5	3 mos.

### Effects of OPT501 treatment on chronic inflammation and Th40 cell levels

**Figure 2:**
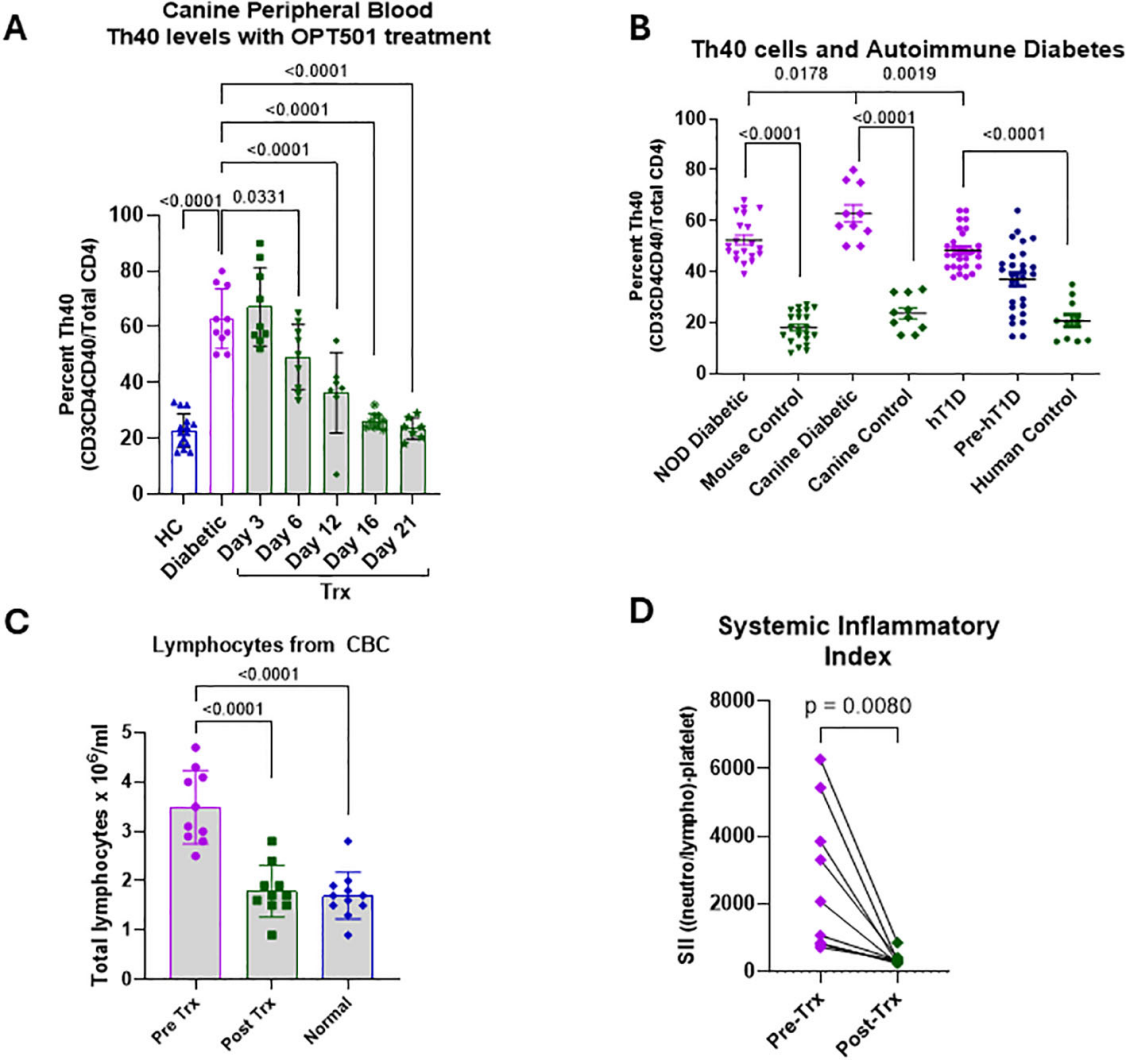
Th40, total lymphocyte, and systemic inflammatory index levels are decreased by OPT501. PBMC were prepared from dogs with diabetes mellitus (CDM; *n* = 10) before treatment and at various times during ongoing OPT501 treatment (Trx) and were **(A)** stained for Th40 cells (CD3^+^CD4^+^CD40^+^; cells were gated on CD3^+^ and then CD4^+^ CD40^+^ within the CD3-gate). Th40 percent of total CD4 is reported. Healthy control (HC; *n* = 11) were included for comparison. **(B)** Th40 levels in NOD diabetic (*n* = 20) and Balb/c mouse controls (*n* = 22), diabetic (*n* = 10) and healthy control (*n* = 10) canines, as well as human type 1 diabetes (hT1D; *n* = 30), pre-diabetic (*n* = 27) and healthy control (*n* = 11) humans are shown. **(C)** Total canine lymphocytes numbers determined from CBC data in CDM dogs (*n* = 10) pre- and post-treatment (Pre-Trx and Post-Trx) compared to healthy controls (HC; *n* = 11). **(D)** Systemic inflammatory index (SII) calculated from CBC data from CDM dogs (n = 10) ( $\frac{neutrphils\;\times \;platelets}{lymphocytes}$). P-value in A is from a one-way ANOVA with Dunnett’s multiple comparisons test. P-values in B-D are from two-tailed t-tests.

CDM subjects were treated as described in methods and **Table 2**. Blood was drawn and PBMC prepared on the first day of treatment prior to administration of OPT501, then at various timepoints throughout the treatment schedule. Healthy control (HC) dogs had mean Th40 cell levels of 22.5%, which is comparable to HC mice and HC humans (no autoimmune disease, no infectious disease, no cancer; **Figure 2A**). Compared to HC dogs, all CDM subjects had significantly elevated Th40 percentages prior to treatment as reported previously (8) (**Figures 2A, B**; p< 0.0001). CDM Th40 percentage was significantly higher than human type 1 diabetes and diabetic NOD mice (**Figure 2B**; p = 0.0019 and p = 0.0178, respectively). After six days of treatment, the Th40 cell percentage began to decline with a further decrease on days 12 and 16 (**Figure 2A**; p = 0.0331, p< 0.0001, and p< 0.0001, respectively). By day 21 the percentage was the same as those of HC dogs (**Figure 2A**; p< 0.0001). Importantly, Th40 cell percentages did not decrease further and remained steady throughout the study.

**Table 2:** Administration and dose for the different routes of treatment with OPT501.

Route of administration	Type of administration	Dose
i.v.	Infusion over 30 minutes	1-2 mg/kg; 3x first week then 1x/week for total of 8 weeks
s.c.	bolus	2mg/kg; 3x first week then 2x/week at 2mg/kg or 1x/week at 4 mg/kg
s.c.	bolus	4 mg/kg; 2x/week

We determined that total lymphocytes in CDM were significantly increased compared to HC (**Figure 2C**; p<0.0001). Treatment with OPT501 reduced total lymphocytes to normal (HC) levels (**Figure 2C**; p<0.0001). We originally reported that CDM dogs experienced a high systemic inflammatory index (SII), which is derived by multiplying the total number of neutrophils and platelets then dividing by the total number of lymphocytes (8). Treatment with OPT501 reduced SII significantly (**Figure 2D**; p=0.0080).

### OPT501 impact on alanine aminotransferase, alkaline phosphatase and cholesterol

**Figure 3:**
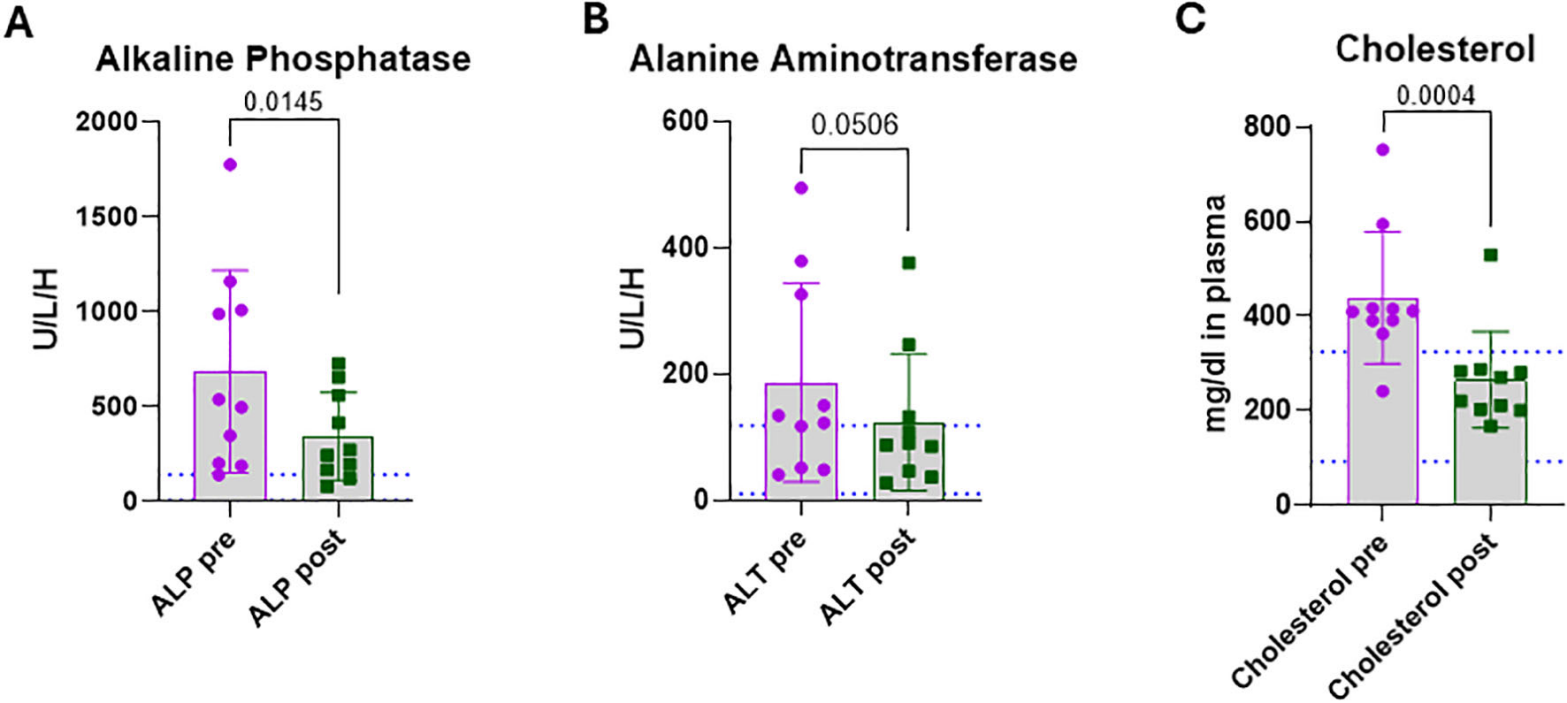
Blood chemistry levels are improved by OPT501 treatment. For chemistry panel, blood was drawn from dogs with diabetes mellitus (n = 10) prior to (pre) and after (post) treatment with OPT501 and chemistry levels are reported. **(A)** Alkaline Phosphatase (ALP). **(B)** Alanine Aminotransferase (ALT). **(C)** Cholesterol. All p-values in **(A–C)** are from two-tailed t-tests.

Alkaline phosphatase (ALP) is an enzyme found in liver, bone, kidney, and intestine (22–24). The primary function is protein breakdown, specifically in liver; in bone it is involved in regulating the mineralization process and Vitamin D metabolism (23). High ALP can reflect liver and/or bone damage, and it is associated with risk of cardiovascular disease including atherosclerosis, dyslipidemia, and hyper-cholesterolemia in humans (25). Alanine Aminotransferase (ALT) converts alanine into pyruvate and produces glutamate, a neurotransmitter. Increases in ALT and ALP are associated with primary and reactive liver disease and are frequently monitored during drug administration as an indicator of hepatotoxicity (26). In the CDM patients, ALP was above the canine normal range in nine of 10 subjects (90%) with four (40%) being >900 U/L/H (**Figure 3A**). ALT was above normal range in six dogs (60%; **Figure 3B**). Following OTP501 administration, ALP was reduced in all dogs, achieving normal range in two (20%) dogs (**Figure 3A**; p = 0.0145). ALT was reduced in all OPT501-treated dogs, with only three remaining out of normal range, however the change did not reach significance (**Figure 3B**; p = 0.0506). These changes suggest that the study drug is not hepatotoxic and positively impacts hepatic changes seen in diabetics.

Elevated serum cholesterol is associated with CDM (27) and cardiovascular disease (CVD) including canine atherosclerosis (28–30). High serum cholesterol is not surprising in human type 2 diabetes but triglyceride and LDL increases can also be associated with human type 1 diabetes (31). Nine of ten CDM subjects (90%) had elevated cholesterol, and the tenth was in the higher half of the normal range (**Figure 3C**). OPT501 treatment reduced cholesterol in all subjects with nine subjects (90%) moving to normal range (**Figure 3C**; p = 0.0004). Although the dogs were not fasted prior to blood collection, a recent study showed no impact of feeding on cholesterol concentrations in healthy dogs (32). The lowered cholesterol outcome suggests an alternate indication for OPT501 in hyper cholesterol associated canine disease.

### OPT501 effect on fructosamine

**Figure 4:**
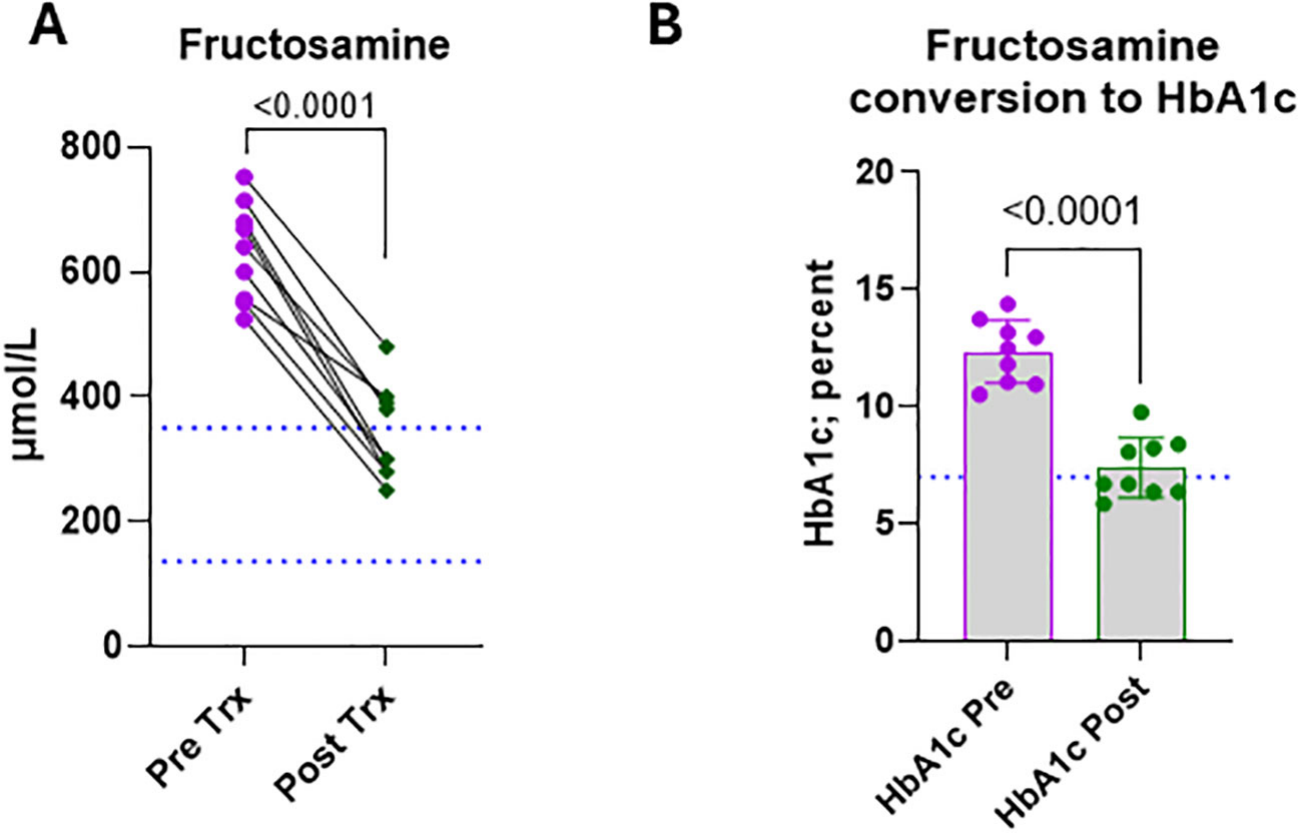
Fructosamine levels are improved by OPT501. Dogs with diabetes mellitus (*n* = 9) were assessed pre- and post-treatment (Trx) for **(A)** glycated fructosamine. Dotted blue lines indicate normal range. **(B)** The values from A were converted to HbA1c equivalent levels for comparison to human Type 1 Diabetes. Dotted blue line indicates the HbA1c target level recommended by the American Diabetes Association, 7% or less. P-values in **(A, B)** are from two-tailed t-tests.

Fructosamine is a compound that forms from glycation reactions between free glucose and primary amines followed by isomerization via an Amadori reaction (33, 34). High fructosamine reflects continuously dysregulated glucose levels and is used as a measure of disease management. Therefore, we compared pre-treatment fructosamine with that measured at study completion. In all CDM subjects, there was an average 1.8-fold reduction in fructosamine over the course of the OPT501 treatment, and five of nine subjects (55.6%) had values in the normal range by the end of the study (**Figure 4A**; p< 0.0001). Using a fructosamine to HbA1c conversion formula (HbA1c = 0.017 × Fructosamine + 1.61) (35), all subjects had HbA1c equivalents above 10% before treatment (**Figure 4B**). OPT501 treatment reduced HbA1c equivalents significantly, with five of nine (55.6%) achieving levels under 7% and three achieving the American Diabetes Association desired goal for human diabetes of 6.5% (**Figure 4B**; p< 0.0001).

### OPT501 treatment reduced plasma glucose as well as insulin requirements

**Figure 5:**
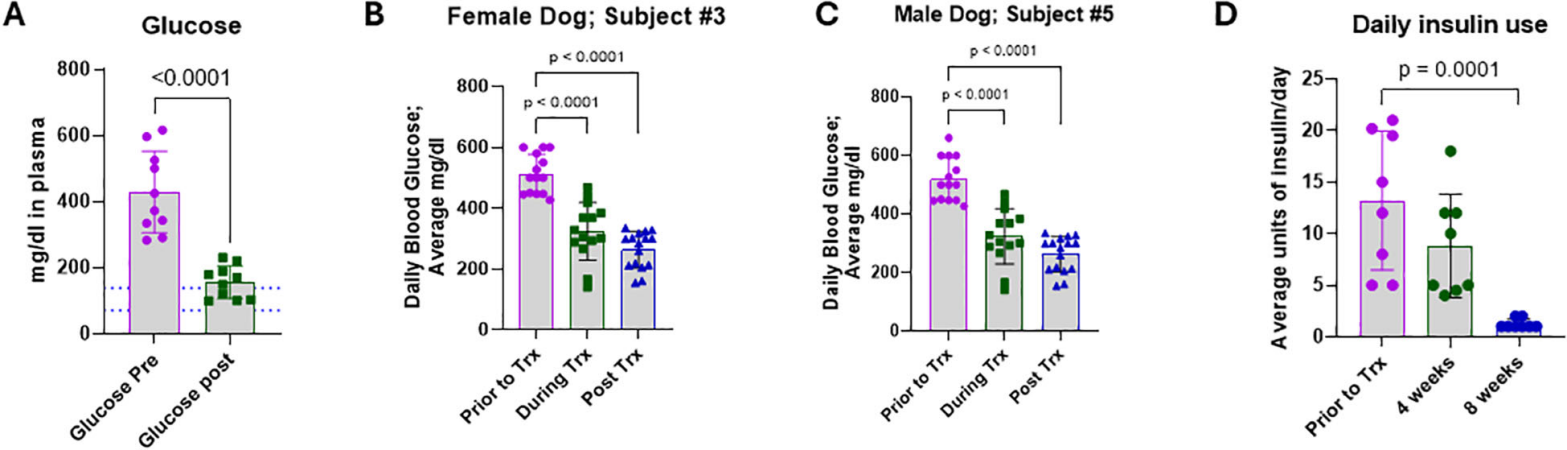
OPT501 controls blood glucose levels and reduces insulin requirements in dogs with diabetes mellitus. **(A)** Blood glucose was measured by standard blood chemistry panel before (Pre) and after (Post) treatment with OPT501 (*n* = 10). Dotted blue lines indicate normal range. **(B, C)** Average daily blood glucose prior to, during, and post-treatment are depicted for a female **(B)** and a male **(C)** dog as measured by a constant glucose monitor. **(D)** Daily average insulin use was recorded prior to, after 4 weeks, and after 8 weeks of OPT501 treatment (*n* = 8). P-value in **(A)** is two-tailed t-test. P-values in **(B–D)** are from one-way ANOVA with Dunnett’s multiple comparisons test.

In all dogs, glucose was measured prior to treatment and post treatment by standard blood chemistry panel. A continuous glucose monitor (CGM) was placed in five of the dogs two weeks prior to treatment and maintained throughout the treatment period. Prior to treatment, all dogs had blood glucose concentrations substantially above normal limits (median: 398 mg/dl, range: 283–616 mg/dl; **Figure 5A**). At the end of treatment all subjects had significantly lower blood glucose, with four of the ten (40%) achieving normal range (**Figure 5A**; p< 0.0001). None of the dogs demonstrated hypoglycemia. Representative CGM data from one female and one male subject are shown (**Figures 5B, C**, respectively). Blood glucose levels were collected for 14 days prior to treatment, during, and post final treatment. Daily averages for the defined periods are shown. In both subjects, OPT501 treatment significantly reduced daily glucose levels that were maintained for two weeks post final dose (**Figures 5B, C**; all p< 0.0001). Daily insulin was recorded by eight owners during the treatment period. Based on consultation with their veterinarian, average daily insulin use was reduced in all subjects by eight weeks (**Figure 5D**; p = 0.0001). Two subjects reduced from 10 U twice daily (20U/day) to 1 U/day; a 95% reduction in insulin.

### OPT501 increased C-peptide levels in CDM

**Figure 6:**
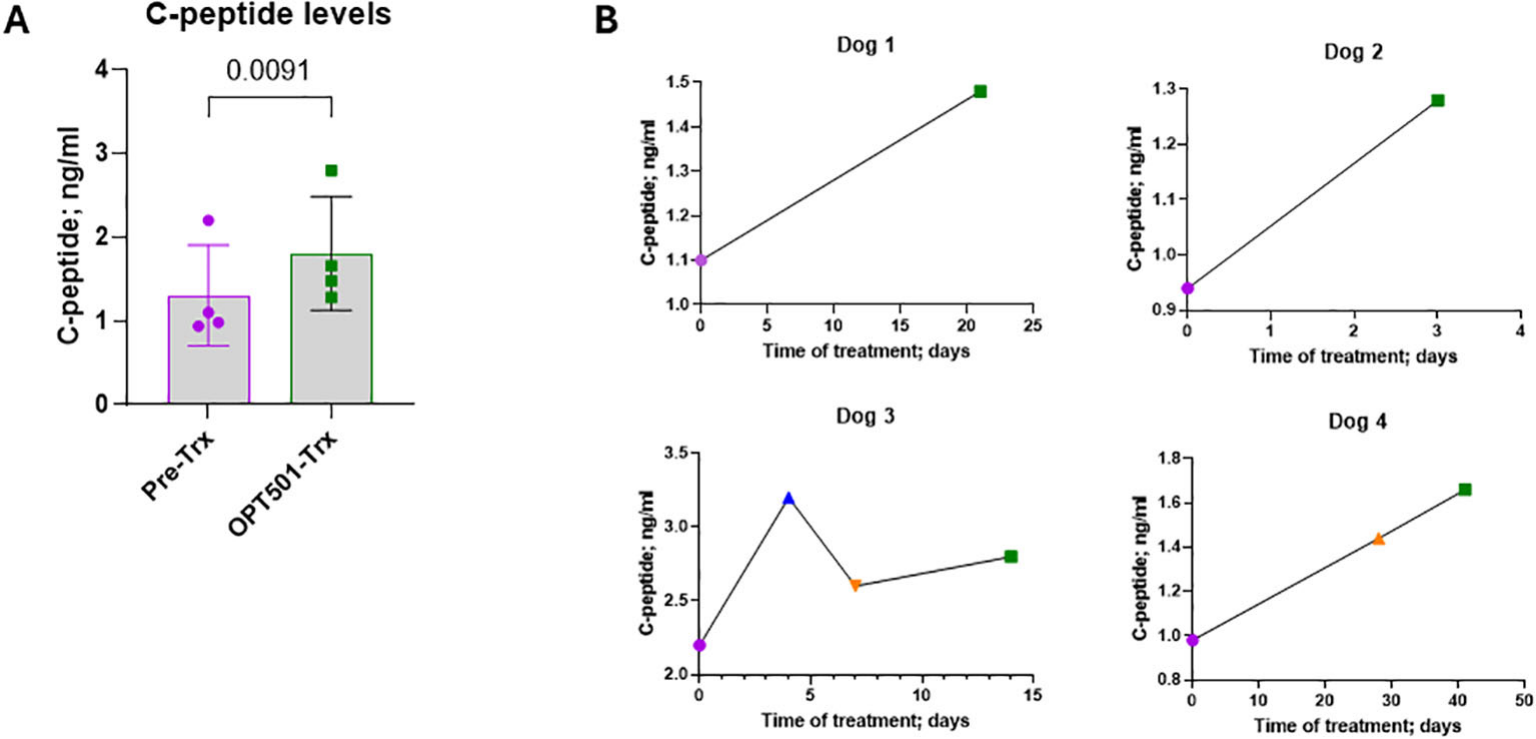
OPT501 increases residual C-peptide levels. **(A)** C-peptide was measured before (Pre) and with OPT501 i.v. treatment (Trx) of dogs with diabetes mellitus (*n* = 4). P-value is from a two-tailed t-test. **(B)** Graphs of individual C-peptide over time for the four dogs.

The American Diabetes Association reports that C-peptide can be detected in up to 29% of human type 1 diabetes, even 31–40 years after disease onset (36), indicating low level residual β-cell function. When disease duration was shorter, the C-peptide values were higher; higher residual C-peptide also was associated with later age at onset (36). With controlled inflammation, β-cell mass regeneration and insulin production could be increased. There are no reports for normal C-peptide levels in canine subjects. C-peptide was measured in four dogs treated with OPT501 by the i.v. route. Two of the four CDM dogs had been diagnosed less than one month and another had been diagnosed 3.5 years prior to study enrollment (**Table 1**). All four dogs had residual levels of C-peptide, and three were low (**Figure 6A**). Insulin and C-peptide have a 1:1 molar ratio in serum (37, 38). When the CDM dogs were treated with OPT501, C-peptide levels increased significantly (**Figure 6A**; p = 0.0091). The individual C-peptide trajectories for each dog are shown (**Figure 6B**).

Anecdotally, most owners (non-blinded) reported that their dogs were more energetic and generally seemed to have improved health while on OPT501 and no owner reported any infections or other adverse events during OPT501 treatment.

## Discussion

This study shows that by modulating CD40 signals an improved level of disease control in CDM can be achieved. OPT501 treatment targeted the known pathogenic effector Th40 cells returning them to levels of HC arguably re-establishing T cell homeostasis. Importantly, the Th40 levels were not ablated, which is a common problem with antibody treatments, where the target cells are subjected to antibody-dependent-cellular-cytotoxicity (ADCC). ADCC often results in lymphopenia and immune suppression with increased risk of infections. The peptide approach affords the recipient a pool of Th40 cells available to fight infections and therefore does not render the recipient at increased risk of immunosuppression. The OPT501 control of overall inflammation was reflected in a concomitant decrease in the systemic inflammatory index, SII, that reflects reductions in platelets and neutrophils. Thus, independent biomarkers for chronic inflammation were reduced by OPT501 treatment in dogs with CDM.

Dysregulated blood glucose levels with glucosuria are major concerns in diabetes regardless of species. Uncontrolled blood glucose spills over in the urine and when glucose cannot be utilized for energy production due to lack of insulin, ketones are generated. Under extreme conditions, diabetic ketoacidosis (DKA) can occur, with decreased pH and electrolyte disturbances that require intensive care (39). In human type 1 diabetes daily glucose levels can be managed relatively well by insulin although only 17-21% of patients achieve HbA1c goals (40). Even with daily insulin dosing, the CDM dogs recruited to this study had daily glucose averages >300 mg/dl and in some cases >450 mg/dl. The difficulty in managing daily glucose in these dogs was reflected by the high fructosamine levels and by the HbA1c levels determined using the conversion equation. Subjects receiving anywhere from 5–20 Units of insulin daily still had glucosuria and some had ketonuria. OPT501 improved daily glucose concentration, eliminated ketonuria, and reduced glucosuria in the recipients. OPT501 significantly reduced fructosamine levels, providing further evidence of the impact of the drug on disease management. Accordingly, regulation of the CD40 inflammatory pathway may help reduce the risk of dogs with CDM developing DKA.

Daily insulin use was significantly lowered in the CDM dogs treated with OPT501 over eight weeks, up to 20-fold lower than at pretreatment. While the C-peptide data are exploratory since the samples were collected without fasting but also not postprandially, the lowered insulin requirement could be explained in part by the fact that OPT501 treated dogs increased their endogenous insulin production by 1.4-fold compared to pretreatment as evident from the C-peptide levels. We also speculate that insulin sensitivity was improved by OPT501 treatment. Since the CDM dogs produced some endogenous insulin (C-peptide) they may simply have started to respond better to that insulin, which in turn required the lowering of exogenous insulin use. Little is known about whether a “honeymoon” defined as a period of remission that is suggested to occur in human subjects, occurs in CDM dogs (41). If that period does occur in CDM, it could explain our C-peptide results. However, anecdotally, dogs are generally diagnosed later after overt disease onset than humans and therefore such a phase may be missed. If a “honeymoon” does exist in CDM, its duration is unknown. CD40 is present on many cell types including adipocytes (42, 43) where it may be involved in insulin resistance (44), therefore it is possible that OPT501 had an impact on adipocytes. Another possibility is that OPT501 treatment upregulated surface expression of glucose transporter 4 (GLUT4) in adipose and muscle tissue, thereby lowering blood glucose. In addition, it is possible that glucotoxicity was improved by OPT501 treatment, which in turn allowed recovery of production, or increase of production, of insulin by beta-islet cells. Although the complete mechanism is not determined, OPT501 treatment resulted in significantly lowered hyperglycemia. In general, C-peptide in CDM is understudied and therefore our data is difficult to put into any larger context.

Limitations of our study include a small sample size in this proof-of-concept study, especially considering the two routes of administration and different doses/dosing schedule. The study started with i.v. administration to evaluate potential feasibility and efficacy and then moved to a more easily administered s.c. route that the dog owners could administer at home. There were no obvious differences between the dose/route groups and therefore all the samples were analyzed together to elucidate any potential OPT501 related changes and reduce alpha statistical error. Because there were no placebo or control groups it is possible that the observed improvements could be influenced by factors such as intensified clinical management, natural disease progression (e.g. a honeymoon period), or regression to the mean. As a pilot study, there was no standardized protocol for diet, physical activity, glucose monitoring etc. While the owners were not instructed to change the dogs’ diets or their physical activity, it is possible that if the dogs began to feel better, related to OPT501 or not, they could increase their physical activity voluntarily, possibly also leading to a change in food intake. Additional limitations include the relatively short duration of treatment, 8–24 weeks. A larger, blinded study, including placebo and/or control groups, of OPT501 administered by the s.c. route compared to current standard of care over a longer period is planned to validate the results and determine how treatment impacts long-term outcomes in dogs with CDM.

OPT501 treatment holds potential as a novel CDM treatment, effective in decreasing inflammation and improving control of disease, resulting in lower blood glucose levels and insulin requirements, and thereby decreasing the risk of developing complications. The data showing that OPT501 lowered serum cholesterol concentrations suggests that there may be alternate indications for this drug that warrant further investigation. Developing OPT501 for routine in home use by clients is the current focus. Given the commonalities between CDM and human type 1 diabetes, including dysregulated Th40 contributing to increased expression of inflammatory cytokines, these results support development of this peptide approach in human type 1 diabetes.

## Data Availability

The original contributions presented in the study are included in the article/**Supplementary Material**. Further inquiries can be directed to the corresponding authors.
