# Supplementary material for: A CD40-targeting peptide, OPT501, modulates inflammation in canine diabetes mellitus improving clinical outcomes
Source: Front Immunol. 2026 Feb 24;17:1759373. doi: 10.3389/fimmu.2026.1759373 (PMC12973222; doi:10.3389/fimmu.2026.1759373)
Supplement: Supplementary file 1 [file DataSheet1.pdf]

Raw data tables

Figure 2A.

HC	Diabetic	Day 0	Day 3	Day 6	Day 12	Day 16	Day 21
15	50	60.5	57.5	33.6	37.2	26.3	20.3
32	80	86	70	65	55	32	18
22	58	78	85	58	38	28	22
25	56	75	90	55	42	26	24
18	62.8	80	78	62	40	24	29
33	50	67	57	50	35	23	24
24	75	75	60	45	7	28	27.5
20	58		55	38		25	
15	76		52	36		24	
32	62.8						
18							
22							
24							
26							
16							

Figure 2B.

Pre-hT1D	hT1D	Human Control	NOD Diabetic	Mouse Control	Canine Diabetic	Canine Control
52	42	12.6	50	25	50	15
22	47.9	20.7	44	24	80	32
23.4	60.7	20	46	27	58	22
53.2	45.2	13.6	48	26	56	25
40.3	64	31.2	52	22	62.8	18
55.8	57	35	68	15	50	33
53.8	46.5	13	65	18	75	24
26.7	49.4	27.5	43	22	58	20
14.6	41.8	22	47	25	76	15
14.5	50	20	65	17	62.8	32
64	47.9	12.5	63	15.5		
26	60.7		58	8		
42.4	37.8		51	12.5		
19.7	64		49	15		
20	57		47	11		
40.8	46.5		39	9		
38.6	49.4		57.5	15		
39	45		48	18		
37.6	38		44.6	22		
42.5	47		64	26		
29.2	42			13.5		
43	39			10		
41.8	45.9					
36	50.2					
28	55					
46	51.6					
47	39					
	41.6					
	48					
	42.2					

Figure 2C.

Pre Trx	Post Trx	Normal
3.1	2.4	2.8
3	1.6	0.9
3.5	2.8	1.9
2.9	0.9	1.7
4	1.9	1.5
4.1	1.7	1.7
4.7	1.5	1.5
4.3	1.7	1.3
2.5	1.5	2
2.8	1.9	1.8
		1.6

Figure 2D.

Pre-Trx	Post-Trx
6278	854
716	334
1070	336
846	246
5435	324
3302	411
801	291
3851	240
2078	246

Figure 3A.

ALP pre	ALP post
537	413
987	652
139	77
495	242
200	165
1159	271
345	193
187	120
1776	726
1008	558

Figure 3B.

ALT pre	ALT post
122	87
379	246
150	108
134	27
40	36
326	90
48	46
117	85

495	376
51	132

Figure 3C.

Chol pre	Chol post
241	220
391	270
415	200
594	211
416	287
411	167
362	202
390	280
409	283
752	529

Figure 4A.

Pre Trx	Post Trx
556	400
669	280
714	380
600	300
752	480
524	250
640	390
550	280
680	300

Figure 4B.

HbA1c Pre	HbA1c Post
11.062	8.41
12.983	6.37
13.748	8.07
11.81	6.71
14.394	9.77
10.518	5.86
12.49	8.24
10.96	6.37
13.17	6.71

Figure 5A.

Glucose Pre	Glucose post
283	169
425	149
290	99
596	188
343	230
500	100
525	103
616	220
334	121

372	178

Figure 5B.

Prior to Trx	During Trx	Post Trx
600	293	211
550	468	335
500	417	300
500	267	205
600	301	328
600	368	284
450	167	259
445	305	155
447	441	161
427	140	324
527	328	300
500	288	218
447	384	213
580	368	299
		317
		301

Figure 5C.

Prior to Trx	During Trx	Post Trx
600	293	211
550	468	335
500	417	300
500	267	205
600	301	328
600	368	284
450	167	259
445	305	155
447	441	161
427	140	324
527	328	300
500	288	218
447	384	213
660	368	299
		317
		301

Figure 5D.

Prior to Trx	4 weeks	8 weeks
20.2	12	1
19.5	18	2
21	10	1
15	12	1
5	5	2
12	5	1
5	4	1
8	4.5	1

Figure 6A.

Pre-Trx	Post-Trx
1.1	1.48
0.94	1.28
2.2	2.8
0.98	1.66

Figure 6B.

Dog 1

**Pre-Trx**	1.1
**Day 21**	1.48

Dog 2

**Pre-Trx**	0.94
**Day 21**	1.28

Dog 3

**Pre-Trx**	2.2
**Day 4**	3.2
**Day 7**	2.6
**Day 14**	2.8

Dog 4

**Pre-Trx**	0.98
**Day 28**	1.44
**Day 41**	1.66
